# *Mesona chinensis* Benth. Extract Ameliorates Hyperlipidemia in High-Fat Diet-Fed Mice and Rats by Regulating the Gut Microbiota

**DOI:** 10.3390/foods13213383

**Published:** 2024-10-24

**Authors:** Huilin Yang, Xiaojuan Song, Xiaofang Huang, Bilian Yu, Cuiqing Lin, Jialin Du, Jiehui Yang, Qing Luo, Jingwen Li, Yinshan Feng, Ruoting Zhan, Ping Yan

**Affiliations:** 1 College of Traditional Chinese Medicine, Guangzhou University of Chinese Medicine, Guangzhou 510006, China; hl.yang0708@outlook.com (H.Y.); m13726838797@163.com (X.S.); huangxiaofang@999.com.cn (X.H.); yubilian@999.com.cn (B.Y.); m15602405844@163.com (C.L.); 1103784059a@gmail.com (J.D.); y02102017@163.com (J.Y.); haruko829@163.com (Q.L.); 15728383696@163.com (J.L.); fys1531128599@163.com (Y.F.); 2 Joint Laboratory of Nation Engineering Research Center for the Pharmaceutics of Traditional Chinese Medicines, Guangzhou 510006, China; 3 Key Laboratory of Chinese Medicinal Resource from Lingnan (Guangzhou University of Chinese Medicine), Ministry of Education, Guangzhou 510006, China

**Keywords:** hyperlipidemia, gut microbiota, glycolipid metabolism, short-chain fatty acid, *Mesona chinensis* Benth

## Abstract

*Mesona chinensis* Benth. (or *Platostoma palustre* (*Blume*) A. J. Paton), an edible and medicinal plant, is the main ingredient in black jelly, Hsian-tsao tea, and beverages, and its processed products are popular in China as well as in Southeast Asian countries. Previous studies have shown that the alcohol extract of *Mesona chinensis* Benth. (MC) can reduce the accumulation of oleic acid and ameliorate hyperlipidemia. However, researchers have not yet determined whether it could improve intestinal permeability and metabolic dysfunction by controlling gut microbial dysbiosis and thus reducing hyperlipidemia. This study aimed to explore the potential mechanism by which MC regulates metabolic function disorders in hyperlipidemic high-fat diet (HFD)-fed rats and mice from the perspective of gut microbiota. This study analyzed the effects of MC on metabolic indices related to hyperlipidemia in HFD-fed rats and the abundance and diversity of the gut microbiota via 16S rRNA V3–4 region pyrosequencing to investigate the regulation of the gut microbiota by MC. We further confirmed that MC ameliorates hyperlipidemia by regulating the gut microbiota by simultaneously administering antibiotics and MC to C57BL/6 mice and measuring their metabolic indices. These results indicate that MC reduces the lipid concentration in the serum of HFD-fed rats, thereby significantly alleviating hyperlipidemia, and regulates the abundance ratio and diversity of the gut microbiota, thereby exerting a beneficial effect on hyperlipidemia. Our further antibiotic experiments in mice revealed that the administration of MC was unable to reduce body weight or serum and organ lipid concentrations in the antibiotic-treated group of hyperlipidemic mice. This study provides evidence that the microbiota is an alternative target for the antihyperlipidemic effect of MC.

## 1. Introduction

Hyperlipidemia is one of the main factors contributing to cardiovascular illnesses. Numerous studies have shown that the global death toll from cardiovascular disease is approximately 17 million per year, and by 2030, it is expected to rise to 23.6 million. Hyperlipidemia is a serious public health problem that endangers both human health and social development. Low levels of HDL-C and high levels of triglycerides (TGs), total cholesterol (TC), and low-density lipoprotein cholesterol (LDL-C) are its defining characteristics. The genesis of hyperlipidemia is attributed to a combination of genetic and environmental variables, including lifestyle choices, nutrition, and dysbiosis of the gut microbiota [1,2].

The gut microbiota is vital to human and animal health and has various physiological functions that can cause several diseases if it is imbalanced. The digestion of meal components and the regulation of fat storage genes by gut bacteria impact the energy homeostasis of the host [3,4]. A high-fat diet (HFD) may produce structural imbalances in the gut microbiota that impede intestinal barrier function and increase endotoxin levels in the bloodstream, which can result in metabolic endotoxemia, insulin resistance, hyperlipidemia, and possibly diabetes [5,6]. Recent evidence from both clinical and experimental research points to the gut microbiota as a possible target for the treatment of hyperlipidemia.

Researchers have been examining how the gut flora is affected by hypolipidemic medications. An imbalance in bacterial structure caused by a high-fat diet can provide insight into the development and incidence of metabolic disorders such as metabolic endotoxemia, hyperlipidemia, and diabetes [7]. The gut microbiota of rats with high cholesterol levels displays dysbacteriosis, characterized by a decrease in Bifidobacteria and Lactobacilli and an increase in Enterobacteria [8]. Traditional lipid-lowering drugs have toxic side effects, and functional foods that prevent and assist in lowering hyperlipidemia are highly valued [9]. 

*Mesona chinensis* Benth. (or *Platostoma palustre* (*Blume*) A. J. Paton) (MC), also known as *Hsian-tsao* in China and Southeast Asian countries, is a popular medicinal and edible plant that is cold in nature and sweet in taste and has the effects of clearing heat, relieving heatstroke, and inducing diuresis. In the Orient, it is also a popular supplement in folklore for heat clearing and is often processed into beverages, black jelly, and tea [10,11,12]. Recent research on *Mesona chinensis* Benth. has revealed that the medicinal effects of this species can improve the lipoprotein profile and atherogenic index of atherosclerotic hamsters and regulate lipid transport and metabolism with hypolipidemic functions [13]. The *Mesona chinensis* Benth. ethanol extract (MC) substantially decreased accumulation in oleic acid (OA)-treated HepG2 cells. It has also been shown that MC can modulate the intestinal microbiota by decreasing the abundances of Lactobacillus and Coprococcus, reversing microbiota dysbiosis caused by dextran sulfate sodium [14]. However, the underlying mechanisms that regulate hyperlipidemia are still poorly understood by people with MC. Whether MC can alleviate hyperlipidemia through gut microbiota is still unknown. Our hypothesis was that MC could treat illnesses related to glycolipid metabolism by changing the composition of the gut microbiota. In this study, we examined the protective effects of MC extract on hyperlipidemic rats and its effects on serum and liver lipids, serum lipid markers, short-chain fatty acids (SCFAs), and the gut microbiota in HFD-fed rats. Furthermore, antibiotics were used to inhibit the intestinal microbiota to verify whether MC can improve glycolipid metabolism disorders by reshaping the intestinal microbiota. The aim of this study was to investigate the potential mechanisms by which MC regulates metabolic dysfunction in hyperlipidaemic high-fat diet (HFD)-fed rats and mice from the perspective of the gut microbiota. These findings lend credence to the notion that a plant-based diet may offer a novel approach to the management and prevention of hyperlipidemia and associated disorders.

## 2. Materials and Methods

### 2.1. Materials and Reagents

All herbs (No. 20140902) were provided by Guangdong Pingyuan Shangju, China, and confirmed by Professor Ruo-Ting Zhan, Guangzhou University of Traditional Chinese Medicine, Guangzhou, China. Simvastatin (No. N030752) was purchased from Hangzhou Merck Pharmaceutical Co., Ltd., Hangzhou, China. Sodium pentobarbital (No. 130315), total cholesterol (TC) (No. A111-1), triglyceride (TG) (No. A110-1), low-density lipoprotein cholesterol (LDL-C) (No. A113-1), and high-density cholesterol (HDL-C) (No. A112-1) assay kits were purchased from Jiancheng Biotechnology Science, Inc. (Nanjing, China).

### 2.2. Preparation of MC Alcohol Extract

The dried powder of *Mesona chinensis* Benth. was mixed with 50% aqueous ethanol solution in a ratio of 1:50 (*m*:*v*) and heated to reflux for 30 min at 90 °C. The mixture was centrifuged at 5000 rpm for 20 min and filtered. The residue was re-extracted under the same conditions and was centrifuged and filtered. All the supernatant was collected, concentrated, and lyophilized to obtain the extract of *Mesona chinensis* Benth. (MC).

### 2.3. High-Performance Liquid Chromatography (HPLC)

The following compounds were created as standard solutions in 50% aqueous ethanol: astragalin, isoquercitrin, rosmarinic acid, caffeic acid, lithospermic acid, and salvianolic acid B. Then, 50% aqueous ethanol was used to extract the dried power of *Mesona chinensis* Benth. at a ratio of 1:50 (*m*:*v*) and heated to reflux for 30 min. The mixtures were filtered by centrifugation, and the residues were re-extracted once under the same conditions. The supernatants of MC alcohol extract were analyzed via Agilent Infinity II 1260 high-performance liquid chromatography. ZORBZX Eclipse XDB-C18 (4.6 × 250 mm, 5 µm) was used for chromatographic separation, with a flow rate of 1 mL/min. Solvent A (acetonitrile) and solvent B (0.2% formic acid) composed the mobile phase. From 5% to 19% solvent A for 0–15 min, 19–21% solvent A for 15–35 min, 21–28% solvent A for 35–40 min, and 28–35% solvent A for 40–55 min covered the linear gradient solution. The separation experiment involved a temperature of 30 °C and a detection wavelength of 320 nm. The HPLC chromatograms of the MC are shown in Supplemental Figure S1.

### 2.4. Animal Experiments

The Animal Ethics Committee of Guangzhou University of Traditional Chinese Medicine approved all the animal experiments. And, after one week of acclimatization, sixty Sprague Dawley rats (Animal Ethics Approval No. 44007200049556) were randomly divided into two main dietary groups: normal diet (NC) (*n* = 10) and high-fat diet (HFD) (*n* = 50). The NC group was fed a normal chow diet, and the HFD group was fed a high-fat chow diet for 8 weeks. Hyperlipidaemic rats were divided into four subgroups after removing rats with failed modeling: the HFD medel group (*n* = 8), the positive control group (PC) receiving 4 mg/kg/day of simvastatin (*n* = 8); the LMC group receiving 2 g/kg/day of MC (*n* = 8); the MMC group (MMC) receiving 4 g/kg/day of MC (*n* = 8); and the HMC group (HMC) receiving 8 g/kg/day of MC (*n* = 8). Due to the discarding of molding failure rats and animal death, only eight rats from each group were finally left for experimental analysis. After 4 weeks of treatment, samples were collected for analysis.

The Animal Ethics Committee of Guangzhou University of Traditional Chinese Medicine approved all the animal experiments. A total of 120 C57BL/6 mice (Animal Ethics Approval No. 44005800011616) were housed in an animal facility designated as pathogen free (SPF) with a light–dark cycle of 12 h, and after one week of acclimatization, the mice were given either a normal diet (NC groups) (*n* = 30) or a high-fat diet (*n* = 90) for 4 weeks. After eliminating the obesity-resistant mice that gained less weight, the high-fat diet-fed mice were further randomly divided into three groups: high-fat diet groups (HFD groups) (*n* = 24), high-fat diet groups (MH groups) receiving 2 g/kg MC per day (*n* = 24), high-fat diet groups (MBH groups) receiving 2 g/kg MC per day, ciprofloxacin (0.2 g/L), metronidazole (1 g/L), and antibiotics (*n* = 24), and a normal group of mice injected with the same volume of saline (*n* = 24). Due to the elimination of mice with modeling failures and the death of animals, only 24 mice per group were ultimately left for experimental analysis.

The oral glucose tolerance test (OGTT) and the insulin tolerance test (ITT) were administered during week twelve of this study. At week 12, the mice were fasted without water overnight, and after the administration of 2 g/kg glucose by gavage, the blood glucose values in the tail-tip vein of the mice were measured via a Roche glucometer at 15, 30, 60, 90, and 120 min, respectively. Blood glucose values were determined at 15, 30, 60, 90, and 120 min after intraperitoneal injection of 0.75 UI/kg insulin (Novalurin R) after 6 h of fasting without water.

**Figure 1:**
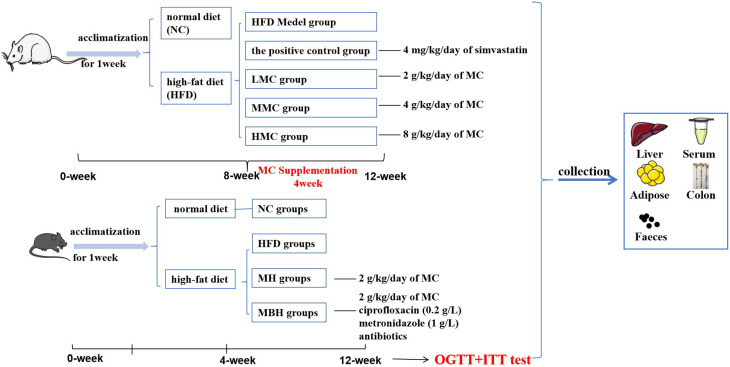
Animal experimental protocol and design.

Two to three days after the end of the OGTT test, stress feces were used, the mice were fixed, and fresh feces were collected in corresponding numbered sterile EP tubes and stored at −80 °C. After fasting for 12 h, the eyeballs of the mice were removed, and blood was collected in sterile 1.5 mL EP tubes (without anticoagulant). After the collection of serum samples, the supernatant was spun at 4 °C and 1500 rpm for 10 min and stored at −80 °C. The supernatant was then stored at −80 °C for 10 min. Organs and tissues such as the liver, intestines, and epididymal fat were stored at −80 °C (Figure 1).

### 2.5. Biochemical Analysis

The blood samples were centrifuged for 15 min at 800 rpm after being incubated for 1 h at room temperature (25 °C) to extract the serum. The collected supernatants were stored at −20 °C. Commercially available enzyme-linked immunosorbent assay kits (Nanjing, China) were used to quantify the serum levels of lipopolysaccharide (LPS), TG, LDL-C, HDL-C, insulin, TNF-α, and IL-6, as well as the contents of TC, TG, apolipoprotein-A1 (Apo-A1), and apolipoprotein B (Apo-B) in the liver homogenate, as directed by the manufacturer. The homeostasis model formula that follows was used to determine the homeostasis model assessment of insulin resistance (HOMA-IR): FBG (mmol/L) × insulin levels (mIU/L)/22.5 is the HOMA-IR.

### 2.6. Histopathological Analysis

Liver sections were subjected to the following H&E staining procedures: fixed, rinsed, dehydrated and fixed, cleared, infiltrated, embedded, trimmed, sectioned, mounted, stained, and photomicrographed.

### 2.7. Gut Microbiota Analysis

The Supplementary Methods provided a detailed description of the procedures used to analyze the taxonomic profiles and diversity of the gut microbiota in each of the sample groups.

### 2.8. Determination of SCFAs

The method for determining the content of SCFAs is described in the Supplementary Methods.

### 2.9. Statistical Analysis

The statistical analysis was carried out via GraphPad Prism 8.0 and SPSS software (version 22.0). One-way analysis of variance and Tukey’s tests were used to identify statistically significant differences. All the data are expressed as the means ± SDs. A statistically significant difference was defined as one with a significance level of *p* < 0.05 between groups.

## 3. Results

### 3.1. MC Intervention Ameliorates Hyperlipidemia-Related Symptoms

**Figure 2:**
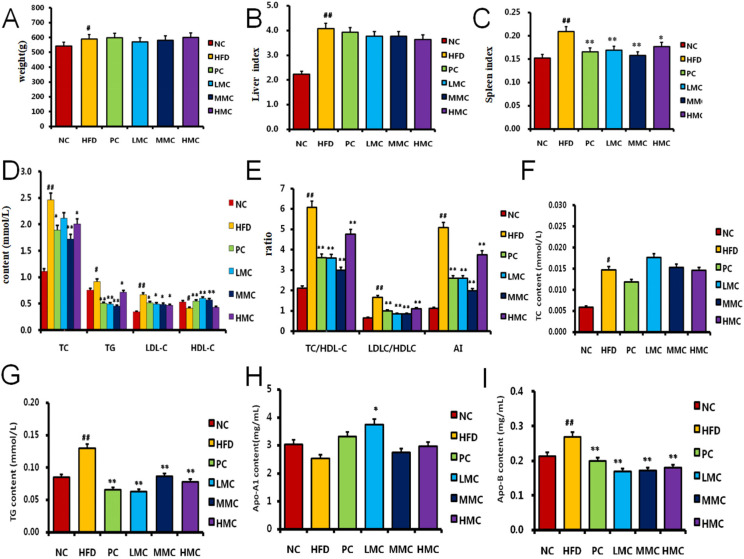
MC intervention ameliorated the hyperlipidemia-related symptoms. (**A**) Body weight, (**B**) liver index, (**C**) spleen index, (**D**,**E**) serum biochemical parameters: serum TC/HDL-C, LDL-C/HDL-C, and AI. (**F**–**I**) Liver biochemical parameters. Significant difference using ANOVA as described in the Methods section. # *p* < 0.05, ## *p* < 0.01 vs. NC; * *p* < 0.05, ** *p* < 0.01 vs. HFD, *n* = 8.

Compared with that in the NC group, there was a discernible increase in weight growth following eight weeks of HFD feeding. However, simvastatin and MC supplementation reduced this increase, and there was no significant difference compared with the HFD group (Figure 2A). Compared with those in the NC group, the liver and spleen indices in the HFD group were considerably greater (*p* < 0.05) (Figure 2). Although no statistically significant difference in liver indices was detected compared with those in the HFD group (*p* > 0.05) (Figure 2B), the spleen indices decreased to varying degrees following MC supplementation (*p* < 0.05, *p* < 0.01) (Figure 2C). Serum and liver biochemical parameters were evaluated to investigate the influence of MC. Serum TC (mmol/L), TG (mmol/L), and LDL-C (mmol/L) levels were significantly elevated in the HFD group (*p* < 0.05), whereas HDL-C (mmol/L) was decreased (Figure 2D). This effect was significantly reversed by MC. Moreover, the atherosclerosis index (AI) [AI = (TC-HDL-C)/HDL-C] in the LMC, MMC, and HMC groups was lower than that in the HFD group. Furthermore, MC significantly reduced the serum TC/HDL-C and LDL-C/HDL-C ratios (*p* < 0.01) (Figure 2E). Compared with the NC diet, the HFD significantly increased the TC, TG, and Apo-B levels in the liver homogenate (*p* < 0.01), indicating that the liver had accumulated fat. However, TG and Apo-B levels were significantly lower in the MC groups (LMC, MMC, and HMC) and the PC group than in the HFD group (*p* < 0.01). No significant difference in the levels of total cholesterol (TC) or Apo-A1 was detected between the MC groups (*p* > 0.05) (Figure 2F–I).

### 3.2. MC Prevents HFD-Induced Liver Steatosis

**Figure 3:**
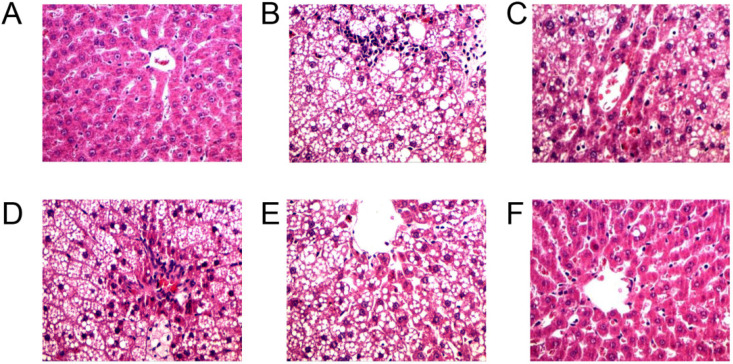
Morphological change in hepatic tissue in high-fat diet-induced hyperlipidemic rats. Few lipid vacuoles were observed in the liver of the NC group (**A**) and PC group (**B**). A low number of cells and high amounts of lipid vacuoles were observed in the HFD group (**C**). The lipid vacuoles decreased, and the number of normal cells increased in the liver of rats treated with LMC group (**D**), MMC group (**E**), and HMC group (**F**).

Hepatic tissue hypertrophy or edema, combined with liver tissue morphology observations and excessive fat intake, leads to fat accumulation, resulting in increased liver burden or lesions and increased liver mass [15]. Pathology-generated liver sections from each group of rats were observed at 200× magnification, as shown in Figure 3. In contrast to those in the HFD group, the cell structures in the NC group were normal, and they exhibited extensive pink staining (Figure 3A). In contrast, the hepatic lobular structure of the liver in the HFD group was destroyed, and the hepatocytes around the central lobular vein of the liver showed noticeable fatty degeneration and gross white coloration (Figure 3B). Nearly every hepatocyte had a wide distribution of lipid dispositions, and HFD-fed animals had more lipid vacuoles than NC-fed mice.

Lipid distribution was significantly reduced in the hepatocytes of the PCs (Figure 3C) and HMC (Figure 3F) of the hyperlipidemic rats after they were treated with MC for four consecutive weeks. The lipid vacuoles were replaced with cytoplasm. No significant differences were observed between LMC (Figure 3D) and MMC (Figure 3E). Like those in the NC and PC groups, the accumulated lipids in the cells nearly disappeared when the MC extract dosage was increased to 8 g/kg, and the degree of liver damage progressively improved (Figure 3F). Thus, compared with the groups treated with MC, the HMC group presented a significant hypolipidemic effect.

### 3.3. MC Regulates SCFAs in Feces

**Figure 4:**
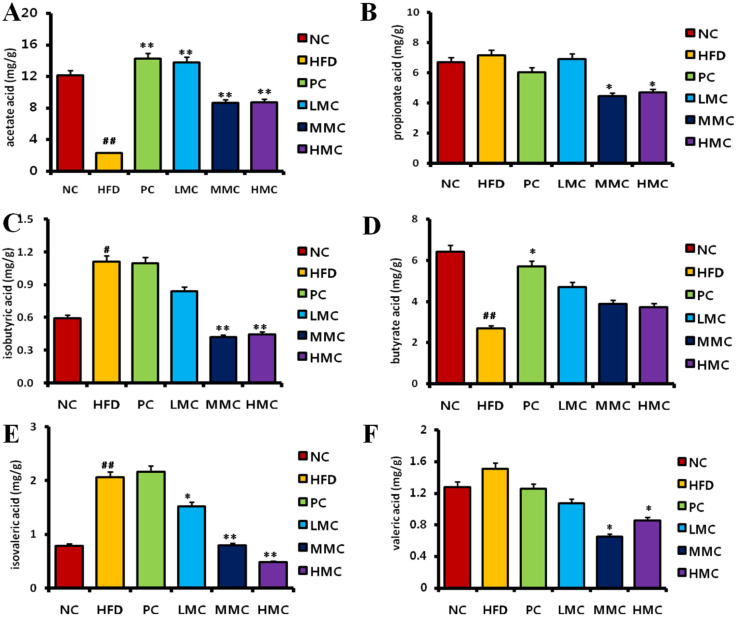
MC regulated FACs in serum and regulated SCFAs in feces. (**A**) Acetate acid content, (**B**) propionate acid content, (**C**) isobutyric acid content, (**D**) butyrate acid content, (**E**) isovaleric acid content, (**F**) valeric acid content. # *p* < 0.05, ## *p* < 0.01 vs. NC; * *p* < 0.05, ** *p* < 0.01 vs. HFD, *n* = 8.

To ascertain the potential regulatory effects of MC, the amounts of SCFAs (acetic acid, propionic acid, butyric acid, isobutyric acid, valeric acid, and isovaleric acid), which are the main microbial fermentation products of meals, were examined in feces. Figure 4 shows that the acetate and butyrate contents in the HFD group were significantly lower (*p* < 0.01) than those in the NC group, whereas the propionic, isobutyric, isovaleric, and valeric acid contents were greater (*p* < 0.05, *p* < 0.01), with isobutyric and isovaleric acids showing the greatest increases. Compared with those in the HFD group, there was a considerable increase in the concentrations of acetic acid (*p* < 0.01) and butyric acid (*p* > 0.05) after MC induction, but the differences were not statistically significant. The MMC and HMC groups presented a significant decrease in the levels of propionic, isobutyric, isovaleric, and valeric acids (*p* < 0.05, *p* < 0.01).

To observe the change in total SCFA levels in each group, we added the SCFA content of the rats in each group (Supplemental Table S1). The results revealed that the HFD group presented the lowest total SCFA content of 16.830 ± 7.057 mg/g. The total SCFA content of the NC, PC, and MC treatment groups was high. The contents of the PC and LMC groups increased to the NC group level (27.904 ± 4.495 mg/g).

### 3.4. MC Modifies the Gut Microbiota Composition in HFD-Fed Rats

**Figure 5:**
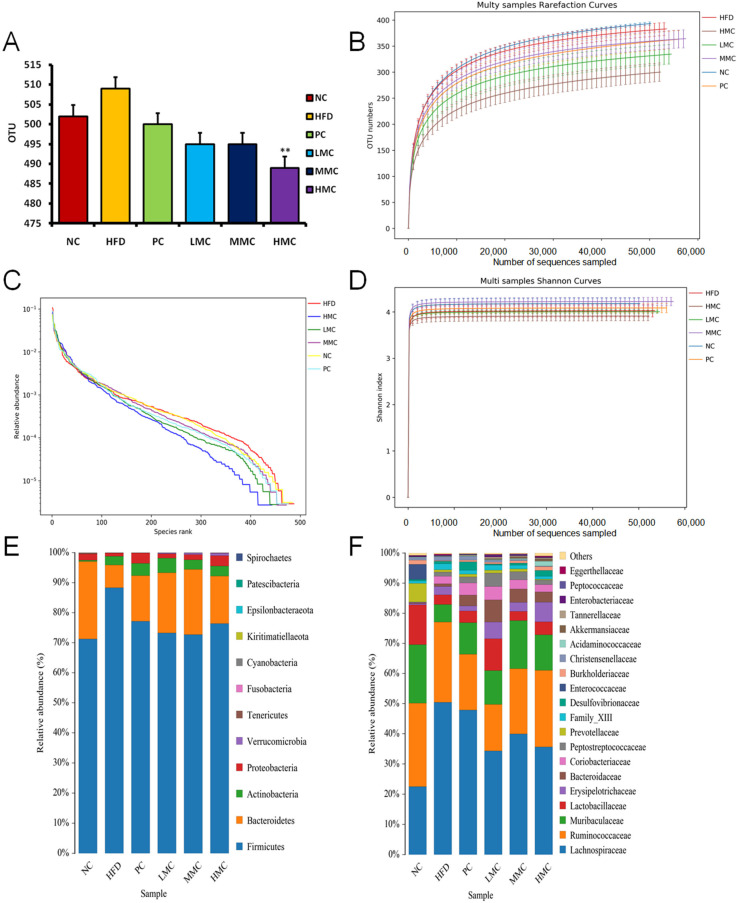
Responses of the diversity, richness, and structure of the gut microbiota to MC during the treatment of hyperlipidemia in rats and (**A**) operational taxonomic unit (OTU) number of gut microbiota in 6 groups; (**B**–**D**) rarefaction curves, rank abundance curve, and Shannon curves of gut microbiota for each sample, respectively. Relative abundances of the gut microbiota at (**E**) the phylum level and (**F**) the family level. ** *p* < 0.01 vs. HFD, *n* = 8.

We used 16S rDNA V3 + V4 sequencing to assess the impact of MC on the composition of the gut microbiota. High-throughput pyrosequencing samples produced 3,831,629 original reads, and after removal of low-quality sequences, 3,389,167 cleaned tags remained, and then, after filtering chimeras, 3,246,950 effective tags were analyzed as follows (Supplemental Table S2). Using a 97% similarity threshold, every successful read was grouped into OTUs. The result shows that the number of OTUs in the HFD group was greater than that in the NC group (Figure 5A). After MC intervention, the number of OTUs decreased, with a significant reduction observed in the HMC group. A rarefaction curve is a curve plotted with the number of sequences as the horizontal coordinate and the corresponding number of detected species as the vertical coordinate, which is used to judge whether the amount and depth of sequencing data can reflect the diversity of species in the samples. The rank abundance curve is a graph of the number of sequences contained in each OTU of the samples in order of size, which can reflect the uniformity of the species contained in the samples, and if the curve tends to flatten as the rank of the species increases, it means that the uniformity of the species composition is higher. The greater the Shannon index, the more species rich the sample OTUs are. When the curve tends to be flat, it means that the amount of sequencing data is large enough, the OTU species will not grow with the increase in sequencing amount, and the diversity index will not change significantly. Species accumμlation curves reflect the relationship between sample size and the number of species annotated. Figure 5B–D show that the rarefaction curve flattens from steep as the sequence increases, indicating that the number of sequences sequenced in this experiment is sufficient to reflect the diversity of the samples tested. The rank abundance curve and Shannon’s index curve of each sample have flattened out, indicating that the diversity of microorganisms distributed evenly in each sample is no longer changing, The results of species accumulation curves show that the accumulation of species in the samples of this experiment tends to be saturated, the species do not increase with the increase in sample size, and the number of samples taken is qualified (Supplemental Figure S2). These findings imply that the sequencing depth is sufficient for further examination.

We subsequently examined each group’s 16S rRNA gene sequences (Supplemental Table S3) to ascertain whether MC influences diversity. We discovered that the greater the Shannon index value was, the lower the Simpson index value was. However, the species diversity was high. These results confirm that HFD feeding can decrease microbial diversity. Moreover, the uniformity and diversity of each group showed the same increasing trend, indicating that MC intervention can increase the number of microbiota.

As shown in Figure 5E and Supplemental Table S4, the proportions of Firmicutes, Bacteroidetes, and Actinobacteria exceeded 99%, whereas the proportions of other bacteria were less than 1%. The phylum-level analysis, as illustrated in Figure 5E, revealed that HFD feeding significantly increased the relative abundance of Firmicutes (88.33% vs. 71.21%) and decreased the relative abundance of Bacteroidetes (7.56% vs. 25.85%). On the other hand, MC therapy increased the ratio of Bacteroidetes to Firmicutes while restoring these levels. Notably, there were no Verrucomicrobia in the NC group, but they were present in the MC group (Supplemental Table S4). MC supplementation significantly increased the abundance of Verrucomicrobia. Furthermore, a family-level analysis was performed to examine the differences among samples (Figure 5F and Supplemental Table S5), which revealed results similar to those observed at the phylum level. Notably, Akkermansiaceae was almost absent in the NC group and HFD group (0.001%) but appeared in the MC administration groups, including the LMC (0.18%), MMC (0.79%), and HMC groups (0.57%). Akkermansiaceae is a probiotic that helps reduce inflammation, hyperlipidemia, and diabetes caused by a high-fat diet. MC supplementation caused a significant increase in these bacteria. These findings suggest that the gut microbiota dysbiosis caused by HFD was reversed by MC therapy.

### 3.5. MC Reduces HFD-Induced Hyperlipidemia-Related Symptoms and Biochemical Parameters via the Gut Microbiota

**Figure 6:**
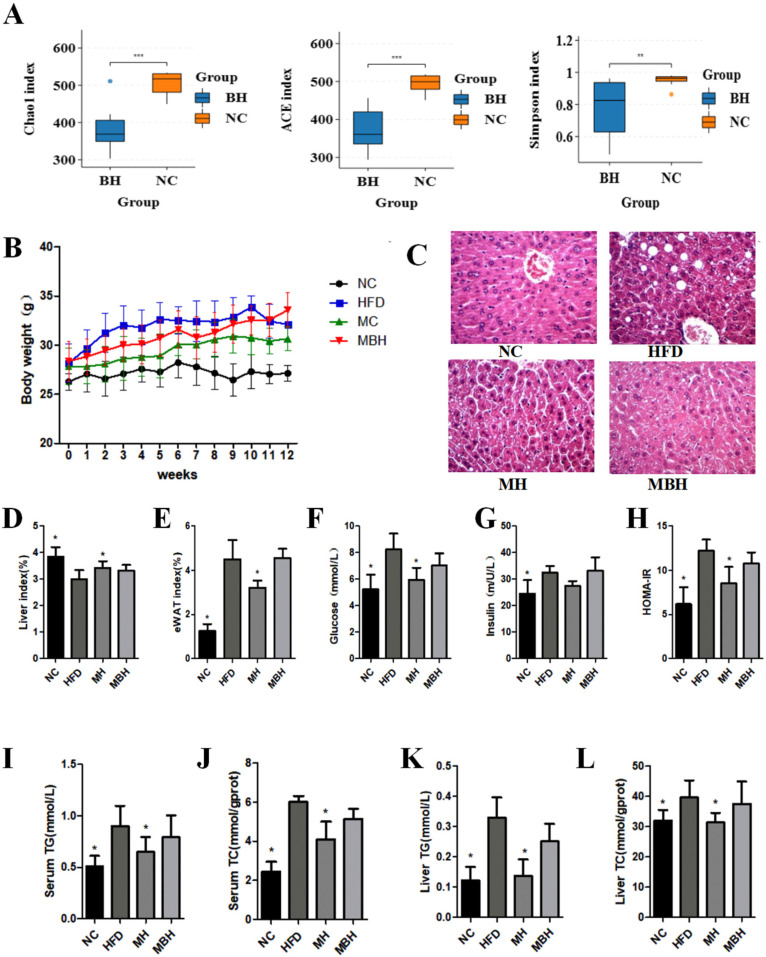
*Mesona chinensis* Benth. (MC) attenuated high-fat diet (HFD)-induced hyperlipidemia. (**A**) Chao index, Ace index, Shannon index, Simpson index of alpha diversity analysis of intestinal microbiota (phylum level); (**B**) changes in the body weight of mice over 12 weeks; (**C**) liver tissue (H&E staining); (**D**) liver index; (**E**) eWAT index; (**F**) glucose; (**G**) insulin; (**H**) homeostasis model assessment (HOMA) of insulin resistance (IR) index; (**I**) serum TG; (**J**) serum TC; (**K**) liver TG; (**L**) liver TC. Data are expressed as mean±standard deviation. One-way ANOVA was used to analyze statistical differences vs. HFD * *p* < 0.05; **: *p* < 0.01; ***: *p* < 0.001.

Previous studies have shown that MC alters the composition of the gut microbiota while treating and preventing hyperlipidemia. Since the bioavailability of MC is extremely low, it is difficult to fully explain whether this is the sole mechanism for its efficacy. Therefore, we hypothesized that the gut microbiota is another target through which MC alleviates hyperlipidemia. To investigate the role of the gut microbiota in the suppression of HFD-induced hyperlipidemia-associated symptoms by MC, a new group of mice was treated with broad-spectrum antibiotics (MBH) for intervention in addition to the NC, HFD, and MC groups. Most gut microbiota are gram-negative bacteria, while ciprofloxacin and metronidazole are broad-spectrum antibiotics for gram-negative bacteria [16]. The results revealed a significant decrease in the Chao1 index, Ace index, and Simpson index in the mice after antibiotic intervention compared with those in the NC group, indicating a significant decrease in the diversity and abundance of the gut microbiota in the mice that received the antibiotic intervention, which confirms the disruptive effect of the antibiotic on the gut microbiota (Figure 6A). The high-fat diet was maintained for 12 weeks, and the body weights and related biochemical parameters were monitored in all four groups of mice. MC significantly attenuated HFD-induced weight gain (*p* < 0.05, Figure 6B), decreased the liver index (*p* < 0.05, Figure 6D), and increased the eWAT index (*p* < 0.05, Figure 6E) while regulating blood glucose levels (*p* < 0.05, Figure 6F), insulin levels (*p* < 0.05, Figure 6G), and the HOMA-IR index (*p* < 0.05, Figure 6H). In contrast, the effect of MC on weight loss was greatly diminished by antibiotic intervention, and there were no significant differences in the liver index, epididymal fat index, blood glucose levels, or insulin levels in the MBH group compared with those in the HFD group. These results suggest that the gut microbiota plays an integral role in the treatment of hyperlipidemic patients with MC. Moreover, the HFD-induced increase in TG and TC in the serum of mice could be effectively reversed by MC intervention, whereas MC did not provide good relief after antibiotic intervention-induced intestinal microbiota disorders (*p* < 0.05, Figure 6I,J). The results of liver HE staining revealed that no inflammatory cells, hepatocellular necrosis, inflammatory cells around the hepatic lobules, or fibrotic changes in the hepatic lobules were observed in the NC group, and the structure of the hepatic lobules was intact. At 12 weeks of the experiment, the HFD group presented obvious fat vesicles, and the hepatocytes of the MH and MBH groups presented slight steatosis, which indicated that MC ameliorated hepatic lipid accumulation in high-fat diet-fed mice (*p* < 0.05, Figure 6C) and that MC reduced the high-fat diet-induced increase in TG and TC in the liver, whereas MBH in mice fed antibiotics did not present the same therapeutic effect (*p* < 0.05, Figure 6K,L). These results suggest that the gut microbiota is an alternative target for MC to alleviate MDS-associated diseases associated with hyperlipidemia.

### 3.6. MC Regulates Metabolites in Obese Mice Through the Gut Microbiota

**Figure 7:**
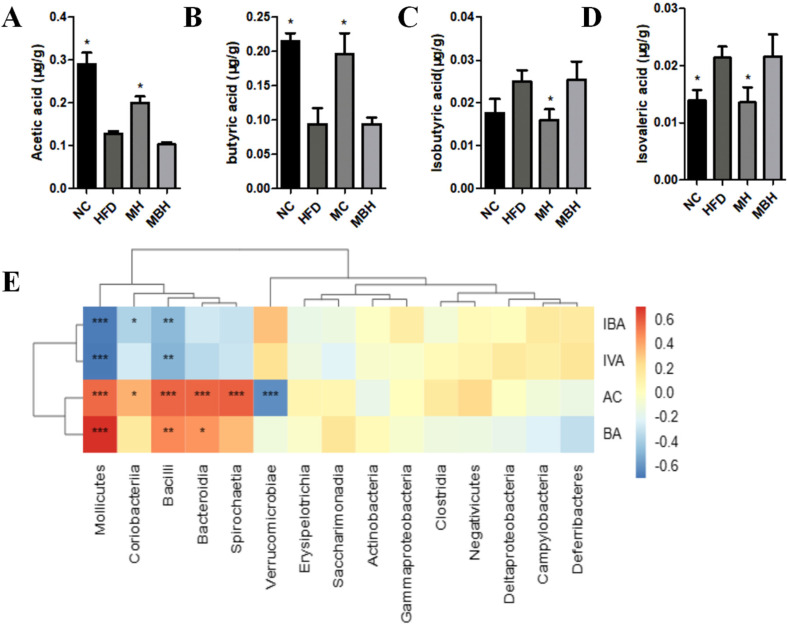
The content of SCFAs and correlation analysis of gut microbiota with SCFAs. (**A**) Acetate acid content, (**B**) butyrate acid content, (**C**) isobutyric acid content, (**D**) isovaleric acid content, (**E**) correlation analysis of gut microbiota with SCFAs. One-way ANOVA was used to analyze statistical differences vs. HFD * *p* < 0.05; **: *p* < 0.01; ***: *p* < 0.001.

The gut microbiota is involved in host metabolism and has a cometabolic relationship with the host. Metabolites produced during fermentation of the intestinal microbiota are also potential mechanisms influencing host metabolism. The results of SCFA detection in feces via gas chromatography revealed a substantial decrease in acetic acid and butyric acid and a significant increase in isobutyric acid and isovaleric acid in HFD-fed mice (*p* < 0.05), which was mitigated by MC supplementation. In the MBH group (*p* < 0.05), MC supplementation did not reverse the changes in SCFA levels caused by the HFD and was not significantly different from that in the HFD group (Figure 7A–D).

A comprehensive understanding of the interactions between the gut microbiota and its metabolites can reveal the beneficial effects of MC. To determine the relationships between the gut microbiota and metabolite parameters, we performed a correlation analysis. The results revealed that fecal acetic acid and butyric acid were positively correlated with *Bacteroidia, Bacilli, Mollicutes,* and Spirochaetes and negatively correlated with *Verrucomicrobiae*, whereas isobutyric acid and isovaleric acid were negatively correlated with *Bacilli and Mollicutes*, which implies that changes in the intestinal flora led to alterations in the fecal SCFA content accordingly (Figure 7E).

## 4. Discussion

Hyperlipidemia, a common condition caused by lipid metabolism disorders, is characterized by considerable increases in the serum levels of TC, TG, and LDL-C, along with substantial decreases in HDL-C concentrations beyond the normal ranges [17]. Compared with the NC group, which was fed a regular diet, the HFD group presented greater serum concentrations of TC, TG, and LDL-C as well as a lower concentration of HDL-C (Figure 2). These data, which are in line with previous research, suggest that a high-fat diet may increase the incidence of hyperlipidemia. High blood lipid concentrations increase the incidence of hyperlipidemia [18]. The serum concentrations of TC, TG, and LDL-C considerably decreased when the rats were fed MC extract, whereas the HDL-C levels increased. Additionally, the MC extract decreased hepatic lipid droplet accumulation, suggesting that it has the ability to lower blood lipid profiles, increase HDL-C levels, and prevent hyperlipidemia through the accumulation of lipid droplets in hepatic tissue cells.

Current medical research has demonstrated a direct relationship between cardiovascular disorders, including atherosclerosis (AS), coronary heart disease (CHD), and hyperlipidemia. Coronary heart disease (CHD) causes AS of the coronary arteries and stenosis or obstruction of the blood vessel lumens, resulting in myocardial hypoxia and ischemia. LDL-C is the main carrier for transporting endogenous cholesterol into peripheral tissue cells, and its concentration is positively correlated with CHD incidence. The results of this study indicated that the serum LDL-C level in the HFD group was considerably greater than that in the NC group; however, the MC extract decreased the LDL-C level in the serum. Additionally, the serum ratios of TC/HDL-C and LDL-C/HDL-C, which are strongly correlated with the severity of CHD, can be utilized as reliable markers to predict the severity of CHD in patients [19]. The TC/HDL-C and LDL-C/HDL-C ratios in the serum were significantly lower in the MC extract groups in this study (*p* < 0.01). Apo-A and Apo-B are the main apolipoproteins of high-density and low-density lipoproteins, respectively. A decrease in Apo-A and an increase in Apo-B are risk factors for CHD [20]. The results of this investigation revealed a significant decrease in the Apo-A1 level in the liver homogenate and an increase in the Apo-B level in the model group. However, the MC extract groups presented increased Apo-A1 levels and significantly reduced Apo-B levels. These findings imply that MC extract might be beneficial for CHD patients. HDL-C elevation can regulate cholesterol reverse transport by transporting cholesterol from extrahepatic tissues to the liver for metabolism, thereby promoting lipid excretion and preventing cholesterol accumulation in extrahepatic tissues or blood vessel walls to cause CHD, AS, and other diseases. The experimental results showed that MC could effectively eliminate high concentrations of TC, TG, and LDL-C by increasing HDL-C levels, thereby maintaining the balance of blood lipid metabolism, accelerating fat decomposition, preventing hyperlipidemia, and regulating blood lipids.

Recently, researchers have focused on the beneficial role of SCFAs in gastrointestinal physiology [21]. Among these SCFAs, acetic acid promotes the secretion of gastric juice, helps digestion, lowers blood fat and cholesterol, dilates blood vessels, and delays blood vessel hardening. The acetic acid concentration was considerably greater in the MC extract groups and significantly lower in the HFD groups. MC extract increases the acetic acid content in rat feces, which can be beneficial for their digestion and can lower blood fat. The liver absorbs propionic acid as a substrate for fat and glycogen production [22]. Excessively produced propionic acid is directly converted to triglycerides in the liver [23], which increases the accumulation of liver fat and may cause fatty lesions in the liver. In this study, the MMC and HMC groups presented a reduced propionic acid content, thereby reducing the accumulation of triglycerides in the body and improving liver fat accumulation. Colonial epithelial cells primarily utilize butyric acid as an energy source [24]. However, it is also connected to hyperlipidemia and plays a role in lipid metabolism by delaying the removal of fat from the intestine [25,26]. Although the difference was not statistically significant, the investigation revealed that simvastatin considerably increased the butyric acid level, which was greater in the MC extract groups than in the HFD group. In this study, the isobutyric and isovaleric acid levels in the HFD group were significantly greater than those in the NC group, which may be related to the increase in protein fermentation. MC intervention effectively reduced isobutyric and isovaleric acid levels and reduced the side effects of protein fermentation. SCFAs provide energy and participate in cholesterol metabolism in the host. The total SCFA content increased in the MC-treated groups, indicating that the reduction in cholesterol levels in MC may be achieved by increasing the excretion of SCFAs in the feces.

Microbial communities regulate host metabolism and energy absorption, which are related to hyperlipidemia [27]. Obese people and animals have been shown to have dysregulated gut microbiota, which is characterized by an increase in Firmicutes and Proteobacteria and a decrease in Bacteroidetes [28,29]. Numerous studies have demonstrated a significant correlation between hyperlipidemia in obese individuals and mice and a greater ratio of Firmicutes to Bacteroidetes. Conversely, a substantial reduction in hyperlipidemia was associated with a low ratio of Firmicutes to Bacteroidetes [30]. Our results confirmed a similar population shift between Firmicutes and Bacteroidetes. Treatment with MC extract reversed these changes, significantly decreased the abundance of Firmicutes, and significantly increased the abundance of Bacteroidetes. Additionally, MC treatment resulted in a reduction in the Firmicutes/Bacteroidetes ratio. Four microbes were increased by MC induction in our study: Akkermansiaceae, Desulfovibrionaceae, Prevotellaceae, and Muribaculaceae; these microbes are considered beneficial bacteria that are commonly more abundant in healthy individuals than in obese people [31,32,33]. As an intestinal symbiont that colonizes the mucosal layer, Akkermansia is thought to be a viable probiotic candidate [34,35]. Recent research has indicated that Akkermansia enhances metabolic health and protects against diabetes, hyperlipidemia, and intestinal inflammation in animals [32,36,37]. MC significantly increased the level of Akkermansia. The lack of Akkermansia is a primary characteristic of hyperlipidemia in humans and HFD-induced mice, which is consistent with the findings of this research. Desulfovibrinaceae is also involved in mucin fermentation and is more abundant in normal-weight children than in overweight children [33]. Prevotellaceae degrades dietary fiber to SCFAs, which serve as the main colonic energy source. Prevotellaceae helps maximize energy extraction from a fiber-rich diet [38]. An imbalance in the gut microbiota is speculated to cause changes in SCFA levels in the feces. The phylum Bacteroidetes is mostly responsible for producing acetate and propionate, whereas the phylum Firmicutes is primarily responsible for producing butyrate, as previously mentioned. It is possible that the abundance of Prevotellaceae, which belongs to the phylum Bacteroidetes, increased, resulting in increases in acetate and propionate in SCFAs.

Since most of the ingested MC passes through the gastrointestinal tract after oral administration of MC, the present study tested the gut microbiota as a therapeutic target for the pharmacological effects of MC, which is a continuation of our studies on the alleviation of HFD-induced hyperlipidemia and an improvement in the composition of the gut microbiota by MC. Thus, on the basis of the experimental results of SD rats, a mouse model of high-fat diet-induced hyperlipidemia was established and divided into four groups, one of which was inhibited by broad-spectrum antibiotics to suppress its bacterial microbiota. The experimental results revealed that, even with MC administration intervention, the phenotypic and biochemical data of hyperlipidemic animals in the inhibition group could not be improved, and the body weights, serum TC, serum TG, and other parameters of the animals in the inhibition group did not significantly differ, demonstrating that the gut microbiota is indeed one of the targets of the antihyperlipidemic effect of MC. This finding is consistent with other findings that alterations in the gut microbiota are important factors associated with hyperlipidemia and hyperlipidemia-induced metabolic disorders [39,40].

Studies have shown that targeting microbiota can interfere with hyperlipidemia [41]. Our study confirms that the gut microbiota is a potential target for MC to ameliorate hyperlipidemia. This is also supported to some extent by changes in the levels of metabolite SCFAs. The results of our study revealed that MC administration in HFD-fed mice significantly reduced the fecal levels of SCFAs, especially acetic acid and butyric acid; however, there was no significant change in the levels of short-chain fatty acids in the MBH group after MC administration. According to the correlation analysis, the intestinal microbiota of mice was correlated with SCFAs, further indicating that the intestinal microbiota plays an important role in the MC-induced regulation of metabolites in HFD-fed mice.

Studies have shown that plant-derived phenolics (including flavonoids, phenolic acids, hydroxyphenols, and catechins) can be effective in preventing and treating hyperlipidemia via the gut microbiota. Nevertheless, previous hyperlipidemia and gut microbiota studies have mostly focused on plant modulation of glycolipid metabolism and modulation of intestinal microbiota in hyperlipidaemic mice (or rats), or one or the other. Furthermore, *Mesona chinensis* Benth. as a popular edible and medicinal plant has been little studied to improve hyperlipidemia, and its potential mechanism to improve hyperlipidemia and gut microbiota is not yet known. In this study, we first showed that MC extract alleviated hyperlipidemia and regulated gut microbiota changes in high-fat diet rats, and then we further confirmed that MC extract could ameliorate hyperlipidemia by modulating the gut microbiota. The experiments conducted in this study provide evidence that the gut microbiota can serve as a therapeutic target for MC.

Previous metabolomics analyses have shown that the active ingredients that produce beneficial effects are usually not phenolics but their metabolites. For example, glycosides, which are the most common natural metabolites, must undergo a first hydrolysis step to produce the more biologically active glycogen form. However, it is unclear whether the antihyperlipidemic and gut microbiota-modulating effects are due to the original metabolites in MC or to their glycosidic forms. SCFAs are important modulators of intestinal homeostasis and host physiological processes, particularly by strengthening the integrity of the intestinal barrier. Since the effects of MC on intestinal barrier morphology and function have not yet been evaluated, whether MC can promote intestinal barrier integrity by regulating SCFAs is not yet known, and the connection and mechanistic role between metabolites and changes in the intestinal flora have not been thoroughly explored and investigated. There are limitations in this study, and the results of this study have not been verified by fecal transplantation experiments. Subsequent experiments should focus on the above topics in further in-depth studies with the goal that subsequent experiments should focus on the above factors in further in-depth research to more deeply interpret the mechanism by which MC improves hyperlipidemia through the gut microbiota.

## 5. Conclusions

This study revealed that MC extract reduced lipid concentrations in the serum and organs, provided hepatoprotection, and ameliorated fecal SCFA abnormalities in high-fat diet-induced hyperlipidemic rats. MC treatment modulated the intestinal microbiota by increasing the abundance of Akkermansiaceae, Desulfovibrionaceae, Prevotellaceae, and Muribaculaceae while restoring the abundance of Firmicutes and Bacteroidetes to normal levels, resulting in beneficial effects on hyperlipidemia. Our in vivo antibiotic experiments in mice further demonstrated that MC administration alleviated the symptoms associated with HFD-induced hyperlipidemia and reversed biochemical parameters, whereas MC administration failed to reduce body weight, serum, or organ lipid concentrations in the antibiotic-treated MBH group of hyperlipidemic mice. Our experimental results demonstrate that MC can ameliorate hyperlipidemia through the microbiota.

Although MC extract can improve hyperlipidemia through the gut microbiota, we were unable to elucidate the mechanism of this association, and further studies are needed to understand whether the improvement in hyperlipidemia is due to specific plant metabolites or a combination of plant metabolites (e.g., flavonoids, phenolic acids, polysaccharides, and terpenoids) in MC, the effect of MC on the morphology and function of the intestinal barrier, and the link and mechanistic role of metabolites with changes in the intestinal microbiota. Moreover, further studies should validate the results using a fecal transplantation assay.

## Data Availability

The original contributions presented in the study are included in the article/Supplementary Material, further inquiries can be directed to the corresponding author.

## Supplementary Materials

The following supporting information can be downloaded at https://www.mdpi.com/article/10.3390/foods13213383/s1. Methods S1: Gut microbiota analysis and Determination of SCFAs; Figure S1: HPLC characteristic chromatogram of MC alcohol extract. Figure S2: species accumulation curves; Table S1: Total short-chain fatty acid changes in rat feces; Table S2: Statistical table of processing results of sequencing data for each rat group; Table S3: Alpha diversity; Table S4: Microbiota at phylum level in each rat group; Table S5: Microbiota at family level in each group.

## Abbreviations

MC	*Mesona chinensis* Benth.
TC	total cholesterol
LDL-C	low-density lipoprotein cholesterol
HDL-C	high-density lipoprotein cholesterol
TGs	triglycerides
HFD	high-fat diet
TC	serum cholesterol
TG	triacylglycerol
OA	oleic acid
SCFA	short-chain fatty acid
NC	normal control group
HFD	high-fat diet group
PC	positive control group
LMC	low-dose Mesona chinensis group
MMC	medium-dose Mesona chinensis group
HMC	high-dose Mesona chinensis group
OGTT	oral glucose tolerance
ITTs	insulin tolerance tests
LPS	lipopolysaccharide
SPF	specific pathogen free
HOMA-IR	homeostasis model assessment of insulin resistance
OTU	operational taxonomic unit
ESI	electrospray ionization
AI	atherosclerosis index
CHD	coronary heart disease
AS	atherosclerosis
