# Supplementary material for: Mesona chinensis Benth. Extract Ameliorates Hyperlipidemia in High-Fat Diet-Fed Mice and Rats by Regulating the Gut Microbiota
Source: Foods. 2024 Oct 24;13(21):3383. doi: 10.3390/foods13213383 (PMC11545744; doi:10.3390/foods13213383)
Supplement: Supplementary file 1 [file foods-13-03383-s001.zip › foods-3257862-supplementary.pdf]

Supplementary Material

1 Supplementary Methods

Gut microbiota analysis

Bacterial genomic DNA was extracted from the frozen stool samples. The thermal cycling for the application of the 16S rRNA of V3–V4 area (338F:5′-ACTCCTACGGGAGG CAGCAG-3′; 806R: 5′-ACTCCTACGGGAGGCAGCAG-3′) was as follows: 3 min initial denaturation at 95 ℃, 27 cycles (30 s at 95 ℃ and 30 s at 55 ℃), 72 ℃ for 10 min. PCR was performed in triplicate. The PCR mixture comprised 10 ng purified template DNA, 2 µL of 2.5 mM dNTPs, 4 µL of 5×FastPfu Buffer, 0.4 µL of FastPfu Polymerase, 0.8 µL of forward and reverse primers (5 µM), 0.2 µL of BSA, and volume up to 20 µL. The paired-end reads obtained by Illumina HiSeq sequencing were spliced according to the overlapping relationship, and the sequences were quality-controlled and filtered. After sequencing, operational taxonomic unit (OTU) cluster and species taxonomy analyses were performed. Based on the results of OTU cluster analysis, OTU was analyzed using various diversity indices and taxonomic information, and statistical analysis of the community structure was carried out at various classification levels. Data were assessed at the phylum and genus levels. Bioinformatics analysis, including intestinal microbiota richness and diversity index (rank-abundance curves), alpha diversity analysis (Chao, Ace, Shannon, and Simpson's indices), beta diversity analysis (principal component analysis [PCA]), species composition analysis, and correlation between the dominant intestinal microbiota and SCFAs (Spearman correlation heatmap), was performed on the Illumina HiSeq sequencing data. To determine whether MC affects diversity, we used the rarefaction curve, rank-abundance curve, Shannon–Wiener curve, alpha diversity, and beta diversity. Rarefaction curves was used to compare the abundance of sample species with different numbers of sequences. Rank-abundance curves was used to explain species abundance and evenness of diversity. The Shannon index curve reflected the microbial diversity in a sample.

Determination of SCFAs

An adequate quantity of frozen mice feces was placed into a 1.5 mL centrifuge tube, accurately weighed, and then mixed with 500 µL of 9.8% sulfuric acid solution and 500 µL of ether. The mixture was vortexed and agitated for 10 min, followed by centrifugation at 12,000 ×g for another 10 min. The resulting supernatant (ether layer) was carefully aspirated, filtered using 0.22-µm organic filter membrane, and placed on ice (volatile) for subsequent injection into the analysis.

SCFAs were detected using a chromatographic column (Agilent DB-FFAP [30 m × 0.250 mm, 0.25 µm] and flame ionization detector (FID) detector. The carrier gas was high-purity nitrogen (purity > 99.999%), and the flow rate was 1.0 mL/min. The temperature of the front injection port was 280 ℃, the injection volume was 1 µL, and the split ratio was 10:1. The gradient heating program was as follows: the initial temperature was 60 °C, increased to 220 °C at 20 °C/min, maintained for 1 min, and then operated at 240 ℃ for 10 min.

2 Supplementary Figures and Tables

2.1 Supplementary Figures

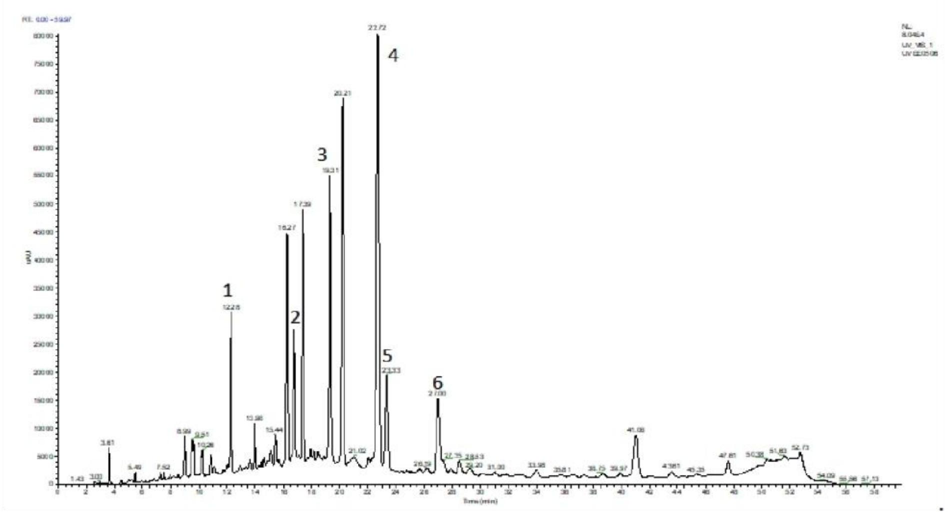

Supplementary Figure S1. HPLC characteristic chromatogram of MC alcohol extract.
Peak 1: caffeic acid; peak 2: isoquercetin; peak 3: astragalin;
peak 4: rosmarinic acid; peak 5: lithospermic acid; peak 6: salvianolic acid B

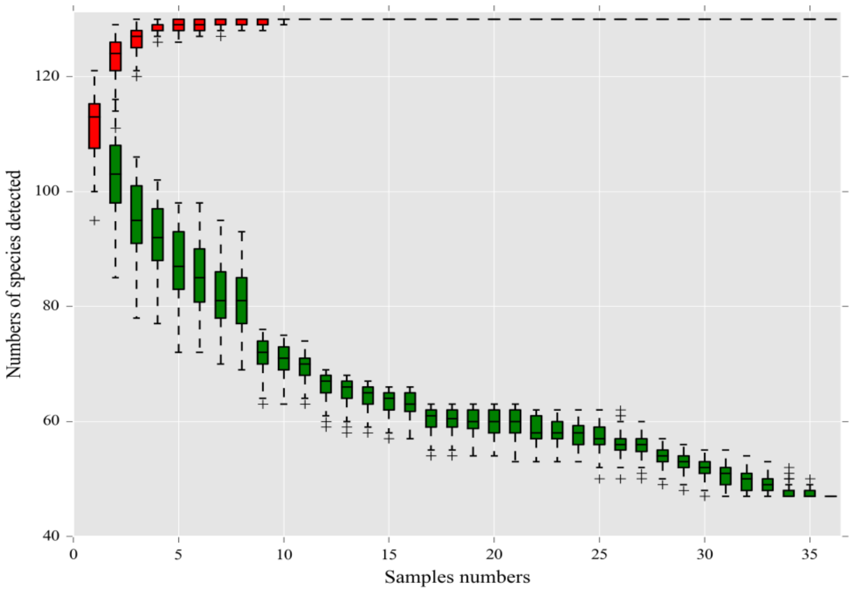

Supplementary Figure S2 species accumulation curves

2.2 Supplementary Tables

Supplemental Table S1 Total short-chain fatty acid changes in rat feces

group	Total short-chain fatty acid（mg/g）
NC	27.904±4.495
HFD	16.830±7.057$^{\#\#}$
PC	30.521±9.759**
LMC	28.849±5.134**
MMC	18.866±5.352
HMC	18.929±6.687

$^{\#}P$< 0.05, $^{\#\#}P$< 0.01vs NC; $^{*}P$< 0.05, $^{**}P$< 0.01vs HFD.

Supplemental Table S2 Statistical table of processing results of sequencing data for each rat group

Sample ID	PE Reads	Clean Tags	Effective Tags	Effective(%)
NC1	79744	69551	67203	84.27
NC2	79899	70054	68377	85.58
NC3	80222	69804	67870	84.6
NC4	80078	70849	65604	81.93
NC5	79930	71660	68695	85.94
NC6	79857	69715	64167	80.35
NC7	80405	71169	67246	83.63
NC8	79943	71206	67781	84.79
HFD1	80046	71677	68854	86.02
HFD2	79904	72273	68194	85.34
HFD3	79991	71231	67607	84.52
HFD4	80137	71912	68747	85.79
HFD5	79985	71517	67339	84.19
HFD6	80253	71901	66434	82.78
HFD7	80168	71983	69062	86.15
HFD8	79755	71150	68029	85.3
PC1	80039	70538	67293	84.08
PC2	80041	70187	66378	82.93
PC3	80026	71036	66927	83.63
PC4	80111	70943	67953	84.82
PC5	80186	71398	66851	83.37
PC6	80097	70639	66601	83.15
PC7	79901	70650	68261	85.43
PC8	79905	70980	67362	84.3

LMC1	80274	71371	70449	87.76
LMC2	79870	69641	63294	79.25
LMC3	80335	71785	68663	85.47
LMC4	80300	70994	67143	83.62
LMC5	79761	70822	67000	84
LMC6	80525	71022	68613	85.21
LMC7	80089	71270	69308	86.54
LMC8	80019	70513	67701	84.61
MMC1	80264	71598	68905	85.85
MMC2	79967	70544	67976	85.01
MMC3	80262	70314	68704	85.6
MMC4	80110	71076	69317	86.53
MMC5	80060	70923	67695	84.56
MMC6	80219	70114	68081	84.87
MMC7	80023	70139	67555	84.42
MMC8	79878	70541	68373	85.6
HMC1	80044	70543	69401	86.7
HMC2	80110	68795	62463	77.97
HMC3	68628	59225	57685	84.05
HMC4	80156	71593	70980	88.55
HMC5	80187	71475	70949	88.48
HMC6	79924	70036	68660	85.91
HMC7	79955	69973	69518	86.95
HMC8	80046	70837	69682	87.05
TOTAL	3831629	3389167	3246950	84.74

Supplemental Table S**3** Alpha diversity

Sample ID	numbers of OTUs	richness index		diversity index		Coverage
		ACE	Chao1	Simpson	Shannon	
NC1	395	423.6	418.6	0.04	4.118	0.99914
NC2	398	435.5	430.2	0.023	4.463	0.99905
NC3	407	425.9	432.5	0.02	4.572	0.9994
NC4	392	414.2	429	0.047	4.078	0.9993
NC5	398	429.8	449.3	0.045	4.089	0.99911
NC6	384	407.7	409.3	0.06	3.762	0.99921
HFD1	349	378.4	394.2	0.05	3.89	0.99931
HFD2	407	422	425	0.022	4.578	0.99948
HFD3	404	436.1	442.1	0.043	4.215	0.99919
HFD4	373	395.4	398.6	0.052	3.901	0.99934
HFD5	420	434.9	436.7	0.082	3.931	0.99948
HFD6	361	382	396.1	0.078	3.638	0.99937
PC1	373	404.9	411.9	0.103	3.676	0.99915
PC2	354	391.9	388.9	0.031	4.199	0.99919
PC3	394	413.4	420	0.028	4.231	0.99933
PC4	393	423	427.1	0.03	4.293	0.9992
PC5	289	330.8	365.2	0.051	3.889	0.99927
PC6	382	407.8	423.1	0.036	4.226	0.99929
LMC1	290	323	326.1	0.043	3.897	0.99921
LMC2	343	354.6	363.3	0.037	4.077	0.99956
LMC3	297	350	352.3	0.041	3.945	0.99915
LMC4	393	406.1	413.3	0.081	3.974	0.99958
LMC5	313	337.7	338.1	0.037	4.009	0.99938
LMC6	391	414.7	424.7	0.038	4.125	0.99935
MMC1	333	350.5	351.1	0.043	4.067	0.99952
MMC2	317	337	338.6	0.031	4.123	0.9995
MMC3	396	422.7	418.5	0.028	4.373	0.99936
MMC4	412	436.8	445.7	0.02	4.567	0.99932
MMC5	339	358	376.2	0.04	3.986	0.99945
MMC6	401	409.9	407	0.031	4.228	0.99965
HMC1	244	285.1	291.1	0.055	3.67	0.99915
HMC2	350	373.2	387	0.027	4.281	0.99927
HMC3	354	380.3	388.2	0.057	3.759	0.99935
HMC4	271	318.9	320.3	0.032	3.962	0.9993
HMC5	309	341.3	346.6	0.054	3.683	0.99934
HMC6	311	344.9	372.5	0.029	4.115	0.99938

Supplemental Table S4 Microbiota at phylum level in each rat group

phylum	NC	HFD	PC	LMC	MMC	HMC
Firmicutes	0.7121	0.8833	0.7709	0.7321	0.7268	0.7634
Bacteroidetes	0.2585	0.0756	0.1520	0.2010	0.2170	0.1587
Proteobacteria	0.0209	0.0110	0.0348	0.0149	0.0170	0.0353
Actinobacteria	0.0039	0.0290	0.0413	0.0483	0.0322	0.0328
Verrucomicrobia	0.0000	0.0000	0.0003	0.0024	0.0058	0.0076
Tenericutes	0.0039	0.0008	0.0001	0.0000	0.0000	0.0000
Fusobacteria	0.0000	0.0000	0.0002	0.0009	0.0003	0.0015
Cyanobacteria	0.0000	0.0000	0.0000	0.0000	0.0005	0.0004
Kiritimatiellaeota	0.0000	0.0000	0.0001	0.0003	0.0001	0.0001
Epsilonbacteraeota	0.0001	0.0000	0.0001	0.0001	0.0002	0.0001
Patescibacteria	0.0004	0.0000	0.0000	0.0000	0.0000	0.0000
Spirochaetes	0.0001	0.0002	0.0000	0.0000	0.0000	0.0000

Supplemental Table S5 Microbiota at family level in each group

family	NC	HFD	PC	LMC	MMC	HMC
Lachnospiraceae	0.24650	0.49672	0.46265	0.34431	0.42795	0.34519
Ruminococcaceae	0.31814	0.25479	0.20725	0.19717	0.18653	0.29399
Muribaculaceae	0.16793	0.04837	0.09550	0.10213	0.13468	0.10364
Lactobacillaceae	0.11582	0.03269	0.03881	0.08730	0.02967	0.05676
Erysipelotrichaceae	0.00424	0.04071	0.02535	0.05118	0.03119	0.05792
Akkermansiaceae	0.00001	0.00001	0.00025	0.00180	0.00785	0.00566
Christensenellaceae	0.01010	0.01174	0.01411	0.00845	0.00352	0.00215
Bacteroidaceae	0.00153	0.01710	0.03064	0.05723	0.04810	0.02559
Coriobacteriaceae	0.00134	0.02913	0.03460	0.04473	0.04547	0.02334
Peptostreptococcaceae	0.00170	0.01904	0.03274	0.03365	0.02318	0.01468
Prevotellaceae	0.05440	0.00585	0.00896	0.01195	0.00746	0.00291
Family_XIII	0.00798	0.01895	0.01232	0.01755	0.01157	0.01038
Desulfovibrionaceae	0.00550	0.00811	0.02130	0.00315	0.02116	0.01519
Burkholderiaceae	0.01119	0.00231	0.00503	0.00462	0.00681	0.01178
Enterococcaceae	0.03744	0.00004	0.00006	0.00004	0.00002	0.00003
Acidaminococcaceae	0.00356	0.00120	0.00145	0.00214	0.00283	0.01333
Eggerthellaceae	0.00211	0.00394	0.00091	0.00289	0.00177	0.00249
Tannerellaceae	0.00012	0.00127	0.00177	0.00426	0.00344	0.00255

Peptococcaceae	0.00152	0.00217	0.00187	0.00204	0.00237	0.00142
Enterobacteriaceae	0.00193	0.00067	0.00101	0.00406	0.00153	0.00180
Streptococcaceae	0.00015	0.00071	0.00030	0.00467	0.00032	0.00090
uncultured_bacterium_o_Mollicutes_RF39	0.00336	0.00119	0.00050	0.00141	0.00001	0.00000
Bifidobacteriaceae	0.00061	0.00013	0.00007	0.00009	0.00007	0.00366
Fusobacteriaceae	0.00000	0.00017	0.00015	0.00195	0.00040	0.00114
Atopobiaceae	0.00042	0.00032	0.00020	0.00063	0.00056	0.00102
Defluviitaleaceae	0.00096	0.00034	0.00052	0.00038	0.00020	0.00043
uncultured_bacterium_o_Coriobacteriales	0.00048	0.00061	0.00034	0.00075	0.00013	0.00014
Leptotrichiaceae	0.00000	0.00000	0.00000	0.00233	0.00000	0.00001
Clostridiaceae_1	0.00013	0.00107	0.00052	0.00008	0.00019	0.00004
Veillonellaceae	0.00003	0.00011	0.00017	0.00141	0.00007	0.00016
Others	0.00080	0.00056	0.00064	0.00565	0.00096	0.00168
